# Clinic-mycological spectrum of *Candida* infection in diabetic foot ulcers in a tertiary care hospital

**DOI:** 10.22034/cmm.2024.345165.1484

**Published:** 2023-12

**Authors:** Azam Moslemi, Tahereh Shokohi, Maryam Salimi, Leila Faeli, Lotfollah Davoodi, Zahra Kashi, Mahdi Abastabar, Iman Haghani, Sabah Mayahi, Seyed Reza Aghili

**Affiliations:** 1 Student Research Committee, Mazandaran University of Medical Sciences, Sari, Iran; 2 Invasive Fungi Research Center, Communicable Diseases Institute, Mazandaran University of Medical Sciences, Sari, Iran; 3 Department of Medical Mycology, School of Medicine, Mazandaran University of Medical Sciences, Sari, Iran; 4 Antimicrobial Resistance Research Center, Communicable Diseases Institute, and Department of Infectious Diseases, Mazandaran University of Medical Sciences, Sari, Iran; 5 Diabetes Research Center, Non-communicable Diseases Institute, Mazandaran University of Medical Sciences, Sari, Iran

**Keywords:** Diabetic foot, Fungal infection, *Candida* infection, *Candida parapsilosis*

## Abstract

**Background and Purpose:**

In diabetic foot ulcers, if fungal agents, such as *Candida* species penetrate the cutaneous or depth of the ulcer, it can increase the wound severity and make it more difficult to heal.

**Materials and Methods:**

A cross-sectional study was performed on 100 diabetic patients with a foot ulcer from December 2019 to November 2020 in northern Iran. Patient data and wound grades were recorded in a questionnaire. *Candida* infection was confirmed by direct microscopic examination and culture. To identify the causative agent, polymerase chain reaction-restriction fragment length polymorphism using *MspI* enzyme and the partial amplification of *hyphal wall proteins* (*HWP1*) gene were performed.

**Results:**

Mean age of the participants was 62.1 ± 10.8 years old, and 95% of them had type 2 diabetes. Moreover, more than 83% of them had diabetes for a duration of 10 years. In addition, 59% of the patients were male, and 66% > of them had poor education levels. Besides, 99% of them were married, and 52% were rural. Furthermore, 95% of the participants had neuropathic symptoms and 88% used antibiotics. The HbA1C level was > 9% in 69% of them, and the mean ulcer grade of the patients was 2.6±1.05. *Candida* infection was detected in 13% of the deep tissue and 7% of the tissue surrounding the wound. The predominant Candida isolate was *C. parapsilosis* (71.5%) and *C. albicans* (14.3%). Infections caused by filamentous fungi were not detected. There was a statistically significant relationship between *Candida* infection and gender, rural lifestyle, HbA1C, and ulcer grade.

**Conclusion:**

Mycological evaluations of diabetic foot ulcers are often ignored. The present study revealed that *C. parapsilosis* is the most common causative agent of deep-seated foot ulcer infection in these patients and may require specific treatment. Therefore, more attention of physicians to *Candida* infections, early diagnosis, and prompt treatment can help accelerate wound healing and prevent amputation.

## Introduction

Diabetes poses a significant global health challenge and places a considerable burden on socioeconomic status. It is estimated that by 2045, the number of diabetic patients will reach approximately 700 million [ 1 ]. It is estimated that 19-34% of diabetic patients suffer from diabetic foot ulcers (DFUs) [ 2 ]. About 56% of DFUs are susceptible to infection, and 20% lead to amputation [ 3 ]. Diabetes affects immune function in ways that increase susceptibility to opportunistic infections. These include decreased T-lymphocyte numbers, decreased neutrophil activity, the release of inflammatory cytokines and antibodies, and increased polymorphonuclear leukocyte death [ 4 ]. 

Fungi are frequently responsible for causing DFU infections, making them a common culprit [ 5 ]. Late identification of fungal infections contributes to severe complications or even fatalities in individuals with diabetes [ 6 ]. Opportunistic fungi, like *Aspergillus* and *Candida* species, are more likely to colonize the skin and foot ulcers than other fungi [ 7 , 8 ]. These infections typically do not show a positive response to antimicrobial treatment, leading to the development of a chronic condition [ 9 , 10 ]. Several studies have reported comparable findings to our research, indicating that *Candida parapsilosis* was the predominant cause of infection in diabetic patients [ 11 , 12 ]. In contrast, Chincholikar et al. reported that the most prevalent fungal isolates in their study was *C. tropicalis* followed by *C. albicans* [ 12 ].

Different Candida species exhibit varying responses to different antifungal medications. A rising concern in clinical settings worldwide is the escalating resistance of *Candida* species to azoles and echinocandins. Previous studies have indicated a significant increase in the prevalence of *C. parapsilosis* as a cause of invasive candidiasis over the past two decades [ 14 ]. Clinicians who manage DFUs often suspect bacterial infections exclusively and administer antibacterial agents as treatment [ 11 ]. The DFU samples are often not sent for fungal culture and sensitivity, resulting in a dearth of literature addressing this concern or supporting the assumption that DFUs are devoid of fungal infections. 

Limited studies in our region have reported the frequency of *Candida* infections in DFUs in recent years. Moreover, many of these studies have not conducted comprehensive molecular investigations to identify the specific *Candida* species responsible for the infections. Therefore, the current study was conducted to evaluate the prevalence of *Candida* infection in DFUs and identify the range of Candida species as causative agents.

## Materials and Methods

### Study population

The present cross-sectional study was performed on 100 patients with DFUs for 12 months, from December 2019 to November 2020, at a diabetes outpatient clinic in an Imam Khomeini hospital affiliated with Mazandaran University of Medical Sciences, Sari, Iran. Information on all patients was collected according to the applicable principles of the Research Ethics Committees of Mazandaran University of Medical Sciences with the ethical code of IR.MAZUMS.REC.1398.1133. 

At the beginning of the study, the informed consent of patients was obtained. Patients with a history of cancer, chemotherapy, or radiotherapy and those who did not agree to participate in this study were excluded. A questionnaire form was designed to record demographic data, medical history, and the duration of diabetes. Moreover, the wound was assessed based on the Wagner Diabetic Foot Ulcer Grade Classification System. [ 15 , 16 ]. In addition, the level of glycosylated hemoglobin (HbA1c) was also recorded in the present study. 

### Specimen collection

**Figure 1:**
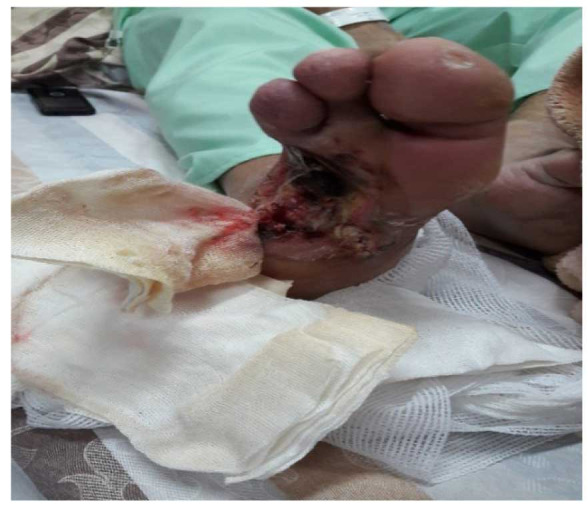
Typical deep ulceration caused by *Candida parapsilosis* in a diabetic patient

Before sampling, the wound area was thoroughly washed by a nurse with a sterile saline solution. Pus and discharge from wounds were collected using sterile swabs, and tissue specimens from the depth of the wound and skin scrapings from the active border areas of lesions were collected with a sterile scalpel (Figure 1). The specimens were immediately transported to the mycology lab for further processing.

### Direct examination

**Figure 2:**
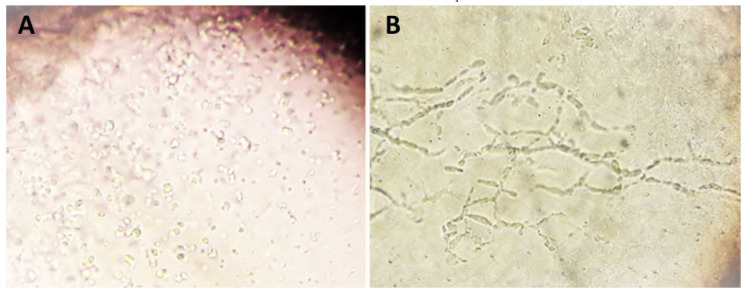
Invasive form of yeast in swab sample of diabetic foot ulcer. Left: blastoconidia, right: pseudohyphae

Direct microscopic examination of specimens for detection of fungal elements (hyphae, pseudohyphae, yeast cells, and blastoconidia) was carried out using a 10% potassium hydroxide preparation (Figure 2). The specimens were inoculated in three media, including Sabouraud Dextrose Agar with chloramphenicol, chloramphenicol and cycloheximide (Merck, Germany), and CHROMagar *Candida* (HiMedia, Mumbai, India). Conventional identification of the grown fungi was performed using the lactophenol aniline blue teased mount and slide culture technique.

### Molecular evaluation

**Figure 3:**
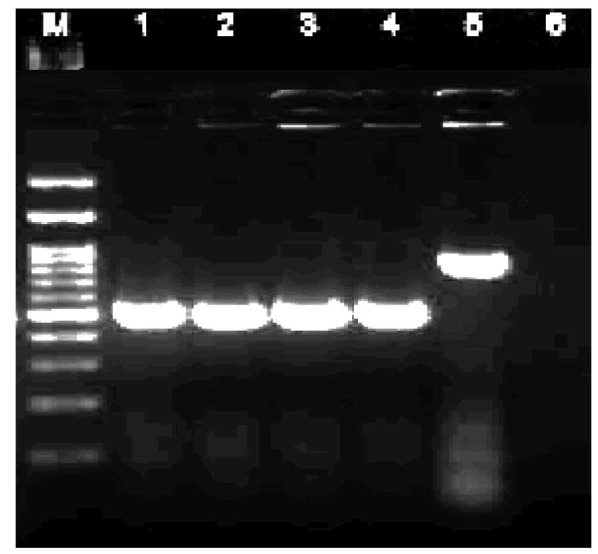
Internal transcribed spacer-polymerase chain reaction products of *Candida* species: Lanes 1-6: *C. tropicalis*, positive control *C. albicans* (CBS 2747), *C. albicans*, *C. parapsilosis*, *C. glabrata*, and negative control. Lane M: molecular size marker

The colony polymerase chain reaction (PCR) was performed with some modifications [ 17 ]. Briefly, each PCR reaction requires 12.5 µl of the master mix and 1 μl of each forward (ITS1: 5’-TAAGTAGGTGTTCCTGCG G-3’) and reverse (ITS4: 5’-TCGTCCGCTTATTCATATGC-3’) primers. A small amount of the colony (approximately 1 mm^3^) was picked and transferred to the microtubes as the DNA template. The following PCR conditions were used to amplify the ITS1–5.8S–ITS4 rDNA region: initial denaturation at 95 °C for 5 min, followed by 35 cycles of denaturation at 94 °C for 30 s, annealing at 56 °C for 1 min, extension at 72 °C for 1 min, and a final extension step at 72 °C for 7 min [ 18 ]. Finally, all amplicons were visualized using 1.5% agarose gel electrophoresis (Figure 3).

**Figure 4:**
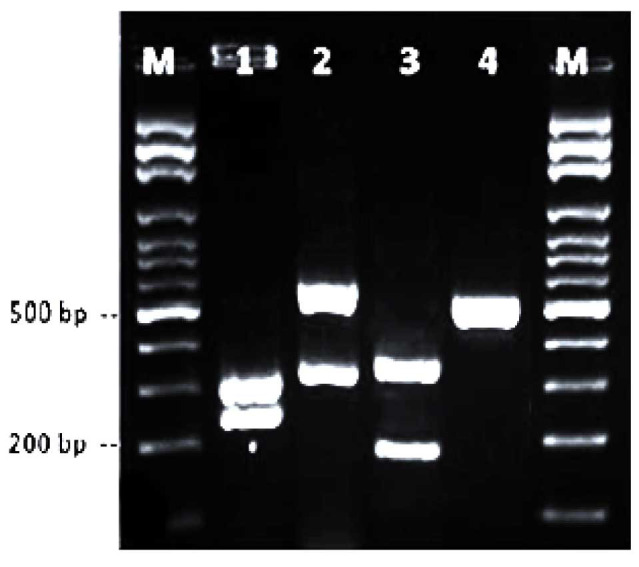
Restriction digestion of polymerase chain reaction products of *Candida* strains with the enzyme *Msp* I. Lanes 1-4: *C. albicans* (298 and 239bp), *C. glabrata* (557 and 314 bp), *C. tropicalis* (340 and 186 bp), and *C. parapsilosis* (520 bp), respectively. Lane M: molecular size marker.

Afterward, PCR-restriction fragment length polymorphism (RFLP) was performed by the restriction enzymes MSPI [ 19 ] to identify common *Candida* species. For this purpose, digestion was performed by incubating 10 μl of each PCR product, digestion buffer (1.5 μl), 5 units of *MSPI* enzyme (Takapouzist, Iran), and dH_2_O up to 15 μl at 37 °C for 2 h. The restriction fragments were visualized by 2% agarose gel electrophoresis (Figure 4).

**Figure 5:**
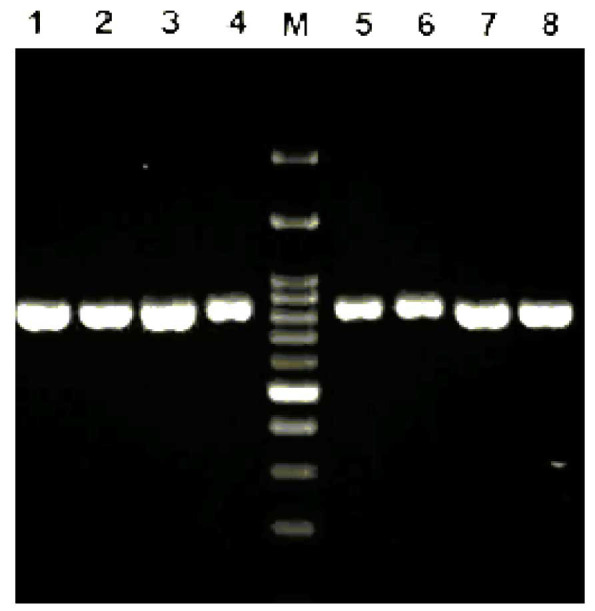
*Candida parapsilosis* polymerase chain reaction product by *HWP*1 gene primers (840 bp), lanes 1-7 our clinical isolates, and lane 8 positive control *C. parapsilosis* (ATCC 90018). Lane M: molecular size marker

In the present study, the *Hyphal Wall Protein* 1 (*HWP*1) gene was used for the identification of the *C. parapsilosis* complex. This process was performed based on the study carried out by Abastabar et al. [ 20 ]. The primer sequence of *C. parapsilosis* was: forward (5'-CGAGGTGAATATGATGCTTGTA-3') and reverse (5'-CCAACAGAATTGCTTAATACCATA-3'). The PCR products of the *HWP*1 gene were visualized by 2% agarose gel electrophoresis (Figure 5).

### Statistical analysis

The statistical analyses were performed in SPSS software (version 18.0), and the data were expressed as mean ± standard deviation (SD).

## Results

Among the 100 patients included in this study, 95% and 5% had type 2 and 1 diabetes, respectively. Moreover, 83% of patients had diabetes for more than 10 years, and 95% of them had symptoms of neuropathy. Mean age of the patients was 62.1 ± 10.8 years (within the range of 30-90 years old). Furthermore, the mean weight and HbA1c of the participants were 76.7 ± 10.9 kg and 9.4 ± 1.8%, respectively. Besides, the mean of wound degree based on the Wagner Classification System was 2.6 ± 1.05. In 13 (13%) patients, *Candida* infection (CI) was identified in the area of the foot ulcer (Table 1).

**Table 1:** Demographic information, duration of diabetes, and medical history of diabetic foot ulcer patients

Value	Sample type	With Candida infection (n=13)	Without Candida infection (n=87)
		n (%)	n (%)
Age (year)	30-40	0 (0)	6 (6.7)
	41-50	0 (0)	11 (12.7)
	51-60	5 (38.45)	22 (25.4)
	61-70	5 (38.45)	35 (40.2)
	71-80	2 (15.4)	11 (12.7)
	81-90	1 (7.7)	2 (2.3)
Gender	Male	11 (84.6)	48 (55.1)
	Female	2 (15.4)	39 (44.9)
Diabetes	Type I	0 (0)	5 (5.74)
	Type II	13 (100)	82 (94.26)
Marital status	Married	13 (100)	86 (98.9)
	Single	0 (0)	1 (1.1)
Occupation	Self-employed	10 (77)	79 (90.8)
	Government employee	3 (23)	8 (9.2)
Literacy	Literate	3 (23)	33 (37.9)
	Illiterate	10 (77)	54 (62.1)
Location	Urban	4 (30.8)	44 (50.57)
	Rural	9 (69.2)	43 (49.43)
Usage antibacterial antibiotics	Yes	12 (92.3)	76 (87.3)
	No	1 (7.7)	11 (12.7)
Usage of corticosteroids	Yes	0 (0)	2 (2.2)
	No	13 (100)	85 (97.8)
Usage of antifungal drugs	Yes	0 (0)	0 (0)
	No	13 (0)	87 (100)
Smoking habits	Yes	1 (7.7)	14 (16.1)
	No	12 (92.3)	73 (83.9)
Alcohol	Yes	0 (0)	0 (0)
	No	13 (100)	87 (100)
HbA1c	<7 %	0 (0)	0 (0)
	>7.1 %	13 (100)	87 (100)
Neuropathy	Yes	13 (100)	82 (94.3)
	No	0 (0)	5 (5.7)
Toenail failure	Yes	10 (77)	71 (81.3)
	No	3 (23)	16 (18.7)
Duration of diabetes	<5	0 (0)	1(1.1)
	5-10	0 (0)	16(18.4)
	>10	13 (100)	70 (80.5)
Foot health	Good	0 (0)	0 (0)
	Moderate	11 (84.6)	67 (77)
	Bad	2 (15.4)	20 (23)
Wagner's wound degree	Grade 1	1 (7.7)	13 (14.9)
	Grade 2	2 (15.4)	33 (38)
	Grade 3	3 (23.1)	29 (33.3)
	Grade 4	4 (30.7)	10 (11.5)
	Grade 5	3 (23.1)	2 (2.3)

None of the DFU patients had a history of taking antifungal drugs. Fungal infections were not detected in any of the subjects under 51 years of age. Results of the present study revealed that the incidence of CI in the foot ulcers of patients was 3.76 times higher in males, compared to females as the CIs were identified in 11 out of 59 males and 2 out of 41 females. In addition, the prevalence of CI in wounds was higher in the illiterate patients, compared to the literate ones. Moreover, its prevalence in rural patients was 2.13 times higher than that in urban residents. It should be noted that CIs had a higher incidence rate in the wounds of patients with sedentary occupations, compared to those with active occupations (27.3% vs. 11.2%). Toenail failure was detected in 77% of diabetic patients with CI; however, it did not yield statistically significant results (*P*>0.05). 

Hypertension, hyperlipidemia, and cardiovascular and renal complications were the most common problems of patients. However, there was no statistically significant difference between the two groups with and without CI in terms of problems and the use of antibacterial antibiotics (*P*>0.05). Usage of clindamycin, alone or in combination with other antibiotics, was more common in patients with CI. Results of the present research showed a significant relationship between increased HbA1C levels and the degree of ulcer and incidence of CI (*P*<0.05). In patients with CI in DFU, the degree of ulcer increased and 10 patients (76.9%) showed degrees higher than 3. Fungal elements were found in 24% of swab samples, 20% of biopsies, and 32% of skin scraping surrounding the ulcers.

The CI was confirmed in 13% of patients by finding fungal elements in an invasive form in preparations of specimens (biopsy, swab, and skin scraps) in direct examination and growth of yeast in culture media. Two species of the genera *Candida*, including *C. parapsilosis* 5 (71.1%) and *C. albicans* 2 (28.9%) were the cause of infection in seven cases of cutaneous candidiasis around the wound. In all patients who were diagnosed with a cutaneous CI around the wound, deep wound infection by the same organism was observed, except for one case where a cutaneous CI was caused by *C. albicans*, but no fungal infection was detected deep inside the wound.

 In this study, no cases of cutaneous or deep DFU infection caused by filamentous fungal agents were detected based on direct examination of samples and the growth of fungal colonies in culture media. However, some filamentous fungi grew in the culture media that were considered contamination or flora. Fusarium spp. (42.8%), *Aspergillus terreus* (14.3%), *Aspergillus flavus* (14.3%), *Paecilomyces* spp. (14.3%), and *Penicillium* spp. (14.3%) were identified.

## Discussion

The DFUs have caused serious financial, psychological, and social challenges for millions of people worldwide. Provision of care for diabetic foot ulcerations is complex and requires multidisciplinary collaboration to provide comprehensive wound care. More than 85% of lower limb amputations in diabetic patients are due to ulcer poly-microbial infections. Until now, many studies have been performed on the prevalence and spectrum of bacterial infections in diabetic wounds, but less attention has been paid to fungal infections [ 16 , 18 ]. 

*Candida* spp. is the most predominantly isolated fungus from these ulcers (less than 5-21%) [ 21 ]. Some studies have reported that among *Candida* species, *C. parapsilosis* is on the top of the list of diabetic lower extremity wounds [ 22 , 23 ], which is similar to the findings of the present research. In some other studies, *C. albicans* was identified as the most common cause of infection in these patients [ 24 - 26 ]. In the present study, it was found that *C. parapsilosis* was the most common infectious agent in 76.9% of the biopsies of wounds and 71.1% of the skin around ulcers. This finding was consistent with those of the studies conducted by Missoni et al. [ 27 ] and Saunte et al. [ 28 ]. However, prevalence of *C. parapsilosis* was low in comparison to the results of a study carried out by Gitau et al. [ 29 ] which showed that *C. parapsilosis* accounted for 6.1% of infections among diabetic patients. 

Moreover, in previous studies, mold species, such as *Aspergillus* spp., *Fusarium* spp., *Penicillium* spp., and *Scopulariopsis* spp. were reported as causative agents of fungal infections in patients with DFU [ 30 - 32 ]. Nevertheless, in the present study, filamentous fungi were isolated from a few samples in culture media but direct examination of the samples did not show the invasive form of the fungi, and these organisms were considered contaminated. Isolation of saprophytic molds in culture media indicates the possibility of their presence in ulcer areas and may cause infection if they penetrate and grow. 

Although dynamic changes in the rates of type 1 and type 2 diabetes in the world are expected over time, type 2 diabetes still accounts for 90% of all diabetes cases [ 33 ]. Studies have shown that DFUs are more common in patients with type 2 diabetes [ 34 , 35 ] and males [ 36 - 38 ]. Moreover, fungal growth has been found to be significantly higher in diabetic patients, especially type 2 diabetics, compared to nondiabetic patients [ 7 ]. In the present study, 95% of all patients as well as the patients with CI had type 2 diabetes. In addition, the frequency of DFU was higher in males, compared to females (59% vs. 41%), and the rate of CI in males with foot ulcers was 3.76 times higher than in females with foot ulcers.

Numerous studies have reported that the majority of patients with DFUs are males above 50 years of age [ 39 - 41 ]. Similarly, results of the present research showed that the majority of patients with DFUs were male and older than 60 years, and that all patients with CI were older than 50 years, the majority of whom (11 out of 13 people) were male. Besides, in this research, similar to other studies [ 25 , 42 ], it was found that although there was no statistically significant difference between the incidence of foot ulcers in diabetic patients and gender, there was a significant difference between the prevalence of CI and gender (*P*=0.0001). However, Gitau et al. performed a study in Kenya and reported a higher proportion of females with DFUs, compared to males and found no significant difference between the incidence of infection and gender [ 29 ]. 

Usage of modern methods has made the identification of the genera and species of the organism more accurate and simpler. By usage of specific primers that anneal at the same temperature and designing PCR products of different sizes, it is possible to detect multiple fungal isolates in a single PCR reaction. This allows for discrimination between different fungal species [ 43 ].

Diabetes can occur at any age, but type 2 diabetes is more common in people over 40 years old and is generally diagnosed in 6-10% of people over 65 years old [ 44 ]. Moreover, the age of patients is one of the factors affecting the incidence of DFUs, and problems in the foot area in diabetics are more common in the age group of 40-86 years old [ 45 ]. Similar to the two-year cohort study performed by Yazdanpanah et al. [ 46 ], the findings of the present study showed that the age groups of 51-60 years old and 61-70 years old had the highest frequency of DFUs and CI. Therefore, ages over 50 can be considered effective factors in the incidence of DFU and fungal infection in this area. Some researchers believe that low is a social and behavioral risk factor associated with ulcers and infection in diabetic patients [ 46 - 48 ]. 

In the present study, although the incidence of foot ulcers was higher in patients with lower education, the incidence of CI in the wound and the level of education did not have a statistically significant relationship. Similar to previous studies [ 46 , 49 , 50 ], the present research showed that most patients with DFUs were married but no statistically significant relationship could be found between marital status and the incidence of foot ulcers. However, Moeini et al. [ 41 ] concluded that wounds were more common in single people. 

Lifestyle of rural patients is different from that of urban patients, and some believe that rural patients are more at the risk of diabetes and foot ulcers. The lower level of hygiene, more contact with the soil, and the possibility of insect bites and damage during agricultural activities in rural areas are risk factors affecting the incidence of wounds in patients [ 51 , 52 ]. Results of the present study also showed that although the frequency of rural patients with DFUs was not significantly different from that of urban patients, the incidence of CI was 2.13 times higher in rural patients, compared to urban patients. 

Intensity and mobility in occupations play an important role in the occurrence of foot ulcers in diabetics [ 53 ]. Findings of the present study revealed that the incidence of ulcers was higher in active occupations, compared to sedentary occupations. However, the frequency of CI in wounds was 2.4 times higher in patients with sedentary occupations, compared to that in patients with active occupations. Therefore, less mobility, followed by poor blood flow to the wound area and prolonged coverage of the foot, can lead to infection. The results showed that lack of hygienic foot care after ulceration is an important factor in causing CI on the surface and depth of the wound. Saeed et al. believe that washing the feet is less effective in fungal infections, although it can prevent other infections [ 54 ]. 

Long duration of diabetes increases the risk of ulcers and infections [ 55 , 56 ]. There is a direct relationship between the duration of diabetes and the incidence of ulcers and infections, especially due to opportunistic organisms, such as Candida spp., and the degree of the ulcer. Usage of antibacterial antibiotics is a precondition for opportunistic yeast infections [ 57 ]. Their use was higher in patients with fungal infections of the foot ulcer; however, due to the high use of these antibiotics in most patients, there was no significant difference between those with and without CI. 

The present study showed that 69.2% of patients with CI in DFUs used clindamycin alone or in combination with other antibiotics, and its use was lower in the group without CI (54%). However, there was no statistically significant difference between these two groups.

Effects of high levels of HbA1C on the cause and development of DFUs are still controversial [ 58 , 59 ], but its effect on wound healing has been proven [ 60 ]. Results of the present study showed that all patients had HbA1C above 7% at the time of sampling; 69% had HbA1C above 9%, and 93% of patients with a CI in DFUs had HbA1C greater than 8%. Some studies have reported that high levels of HbA1C are directly related to fungal infections [ 35 , 40 , 61 ]. 

Grade 2 and 3 ulcers were more common among all patients (67%). Moreover, 76.9% of patients with CI in ulcers had an ulcer degree of ≥ 3. Infiltration and development of fungal agents in the ulcer cause more inflammation and an increase in the degree of the ulcer in patients, and consequently, the treatment will be more difficult and expensive.

In a direct examination by microscopic observation to detect CI in the superficial and deep area of the ulcers, the biopsy and cutaneous scraping specimens are of greater predictive value than the swab specimen. In the present study, 13% of CIs were detected in the depth of ulcers, which was associated with cutaneous candidiasis around the ulcers in six cases (46.2%). Raiesi et al. [ 62 ] in their study performed in Isfahan, Iran, reported the prevalence of CI in DFUs at 19%.

## Conclusion

This study aimed to determine the prevalence of CI in DFUs. The findings indicated that CIs were observed in more than 13% of patients with DFU and *C. parapsilosis* was the predominant agent. Therefore, the evaluation of ulcers for fungal infections is essential. It is concluded that preparation of a biopsy of the ulcer tissue and the scraping of skin around the wound, performance of direct microscopic examination, and culture of the samples would be a good way to determine fungal infections. Therefore, a molecular method, such as PCR-RFLP followed by the use of *HWP*1 gene primers is one of the most valid methods that can be cited which enables us to identify the agent of CI. However, this study was performed during the COVID-19 virus pandemic which led to fewer visits by diabetic patients to medical centers due to the fear of contracting the disease. Therefore, further research with a larger sample size is required to evaluate the clinic-mycological spectrum of fungal infection in DFUs.

## References

[1] Gentile S, Strollo F, Corte TD, Marino G, Guarino G, Italian Study Group on Injection T. ( 2018). Skin complications of insulin injections: A case presentation and a possible explanation of hypoglycaemia. Diabetes Res Clin Pract.

[2] Armstrong D, Boulton A. M. ( 2017). Bus SA Diabetic foot ulcers and their recurrence. N Engl J Med.

[3] Mehra BK, Singh AK, Gupta D, Narang R, Patil R ( 2017). A Clinicomicrobiological study on incidence of Mycotic infections in diabetic foot ulcers. International Journal of Scientific Study.

[4] Casqueiro J, Casqueiro J, Alves C ( 2012). Infections in patients with diabetes mellitus: A review of pathogenesis. Indian J Endocrinol Metab.

[5] Hassan MA, Tamer TM, Rageh AA, Abou-Zeid AM, Abd El-Zaher EH, Kenawy E-R ( 2019). Insight into multidrug-resistant microorganisms from microbial infected diabetic foot ulcers. Diabetes Metab Syndr.

[6] Clayton Jr W, Elasy TA ( 2009). A review of the pathophysiology, classification, and treatment of foot ulcers in diabetic patients. Clinical diabetes.

[7] Singh N, Armstrong DG, Lipsky BA ( 2005). Preventing foot ulcers in patients with diabetes. JAMA.

[8] Raiesi O, Shabandoust H, Dehghan P, Shamsaei S, Soleimani A ( 2018). Fungal infection in foot diabetic patients. J Basic Res Med Sci.

[9] Kamalzadeh S, Sabokbar A ( 2014). Molecular survey of pathogen species of Aspergillus isolated from diabetic foot lesion using nested PCR method. Journal of Torbat Heydariyeh University of Medical Sciences.

[10] Ross-Flanigan N ( 2002). Antifungal Drays Systemic. Qale Encyaopedia Of Medicine.

[11] Chellan G, Shivaprakash S, Karimassery Ramaiyar S, Varma AK, Varma N, Thekkeparambil Sukumaran M, et al ( 2010). Spectrum and prevalence of fungi infecting deep tissues of lower-limb wounds in patients with type 2 diabetes. J Clin Microbiol.

[12] Rodrigues CF, Rodrigues ME, Henriques M ( 2019). Candida sp. infections in patients with diabetes mellitus. J Clin Med.

[13] Chincholikar DA, Pal RB ( 2002). Study of fungal and bacterial infections of the diabetic foot. Indian journal of pathology & microbiology.

[14] Trofa D, Gacser A, Nosanchuk JD ( 2008). Candida parapsilosis, an emerging fungal pathogen. Clin Microbiol Rev.

[15] Mirhendi H, Diba K, Rezaei A, Jalalizand N, Hosseinpur L, Khodadadi H ( 2007). Colony PCR is a rapid and sensitive method for DNA amplification in yeasts. Iran J Public Health.

[16] Noor S, Zubair M, Ahmad J ( 2015). Diabetic foot ulcer--A review on pathophysiology, classification and microbial etiology. Diabetes Metab Syndr.

[17] Mohammadi F, Javaheri MR, Nekoeian S, Dehghan P ( 2016). Identification of Candida species in the oral cavity of diabetic patients. Curr Med Mycol.

[18] Bansal E, Garg A, Bhatia S, Attri AK, Chander J ( 2008). Spectrum of microbial flora in diabetic foot ulcers. Indian J Pathol Microbiol.

[19] Fata S, Saeed Modaghegh MH, Faizi R, Najafzadeh MJ, Afzalaghaee M, Ghasemi M, et al (2011). Mycotic infections in diabetic foot ulcers in Emam Reza hospital, Mashhad, 2006-2008. Jundishapur J Microbiol.

[20] Abastabar M, Hosseinpoor S, Hedayati MT, Shokohi T, Valadan R, Mirhendi H, et al ( 2016). Hyphal wall protein 1 gene: A potential marker for the identification of different Candida species and phylogenetic analysis. Curr Med Mycol.

[21] Musyoki V ( 2020). Speciation and antifungal susceptibility of Candida species isolated from diabetic foot ulcer patients in a tertiary hospital in Kenya. Int J Infect Dis.

[22] Arun CS, Raju P, Lakshmanan V, Kumar A, Bal A, Kumar H ( 2019). Emergence of Fluconazole-resistant Candida Infections in Diabetic Foot Ulcers: Implications for Public Health. Indian J Community Med.

[23] Saseedharan S, Sahu M, Chaddha R, Pathrose E, Bal A, Bhalekar P, et al ( 2018). Epidemiology of diabetic foot infections in a reference tertiary hospital in India. Braz J Microbiol.

[24] Abilash S, Kannan N, Rajan K, Pramodhini M, Ramanathan M ( 2015). Clinical study on the prevalance of fungal infections in diabetic foot ulcers. International Journal of Current Research and Review.

[25] Saud B, Bajgain P, Paudel G, Shrestha V, Bajracharya D, Adhikari S, et al ( 2020). Fungal infection among diabetic and nondiabetic individuals in Nepal. Interdiscip Perspect Infect Dis.

[26] Rodrigues CF, Rodrigues ME, Henriques M ( 2019). Candida sp. Infections in Patients with Diabetes Mellitus. J Clin Med.

[27] Missoni EM, Kalenic S, Vukelic M, De Syo D, Belicza M, Kern J, et al ( 2006). [Role of yeasts in diabetic foot ulcer infection]. Acta Med Croatica.

[28] Saunte DM, Holgersen JB, Haedersdal M, Strauss G, Bitsch M, Svendsen OL, et al ( 2006). Prevalence of toe nail onychomycosis in diabetic patients. Acta Derm Venereol.

[29] Gitau A, Sigilai W, Bii C, Mwangi M ( 2011). Fungal infections among diabetic foot ulcer-patients attending diabetic clinic in Kenyatta National Hospital, Kenya. East African Medical Journal.

[30] Eckhard M, Lengler A, Liersch J, Bretzel RG, Mayser P ( 2007). Fungal foot infections in patients with diabetes mellitus--results of two independent investigations. Mycoses.

[31] Nair S, Peter S, Sasidharan A, Sistla S, Unni AKK ( 2006). Incidence of mycotic infections in diabetic foot tissue. Journal of culture collections.

[32] Taj-Aldeen SJ, Gene J, Al Bozom I, Buzina W, Cano JF, Guarro J ( 2006). Gangrenous necrosis of the diabetic foot caused by Fusarium acutatum. Med Mycol.

[33] Chen L, Magliano DJ, Zimmet PZ ( 2012). The worldwide epidemiology of type 2 diabetes mellitus—present and future perspectives. Nat Rev Endocrinol.

[34] Al-Rubeaan K, Al Derwish M, Ouizi S, Youssef AM, Subhani SN, Ibrahim HM, et al ( 2015). Diabetic foot complications and their risk factors from a large retrospective cohort study. PloS one.

[35] Lane KL, Abusamaan MS, Voss BF, Thurber EG, Al-Hajri N, Gopakumar S, et al ( 2020). Glycemic control and diabetic foot ulcer outcomes: A systematic review and meta-analysis of observational studies. J Diabetes Complications.

[36] Zhang P, Lu J, Jing Y, Tang S, Zhu D, Bi Y ( 2017). Global epidemiology of diabetic foot ulceration: a systematic review and meta-analysis (dagger). Ann Med.

[37] Deribe B, Woldemichael K, Nemera G ( 2014). Prevalence and factors influencing diabetic foot ulcer among diabetic patients attending Arbaminch Hospital, South Ethiopia. J Diabetes Metab.

[38] Shahi SK, Singh VK, Kumar A, Gupta SK, Singh SK ( 2013). Interaction of dihydrofolate reductase and aminoglycoside adenyltransferase enzyme from Klebsiella pneumoniae multidrug resistant strain DF12SA with clindamycin: a molecular modelling and docking study. J Mol Model.

[39] Sanniyasi S, Balu J, Narayanan CD ( 2015). Fungal Infection: A hiddenenemy in Diabetic Foot Ulcers. The J Foot and Ankle Surgery (Asia Pacific), July-Dec.

[40] Akkus G, Evran M, Gungor D, Karakas M, Sert M, Tetiker T ( 2016). Tinea pedis and onychomycosis frequency in diabetes mellitus patients and diabetic foot ulcers. A cross sectional - observational study. Pak J Med Sci.

[41] Moeini M, Shahriari M, Yousefi H, Esfandiari J, Babaahmadi M ( 2017). An investigation on the wound severity and its association with predisposing factors in patients with diabetic foot. J Clin Nurs Midwifery.

[42] Pailian H, Carey SE, Halberda J, Pepperberg IM ( 2020). Age and species comparisons of visual mental manipulation ability as evidence for its development and evolution. Sci Rep.

[43] Atkins SD, Clark IM ( 2004). Fungal molecular diagnostics: a mini review. J Appl Genet.

[44] Dowd SE, Delton Hanson J, Rees E, Wolcott RD, Zischau AM, Sun Y, et al ( 2011). Survey of fungi and yeast in polymicrobial infections in chronic wounds. J Wound Care.

[45] Pataky Z, Golay A, Rieker A, Grandjean R, Schiesari L, Vuagnat H ( 2007). A first evaluation of an educational program for health care providers in a long-term care facility to prevent foot complications. Int J Low Extrem Wounds.

[46] Yazdanpanah L, Shahbazian H, Nazari I, Arti HR, Ahmadi F, Mohammadianinejad SE, et al ( 2018). Prevalence and related risk factors of diabetic foot ulcer in Ahvaz, south west of Iran. Diabetes Metab Syndr.

[47] Banik PC, Barua L, Moniruzzaman M, Mondal R, Zaman F, Ali L ( 2020). Risk of diabetic foot ulcer and its associated factors among Bangladeshi subjects: a multicentric cross-sectional study. BMJ open.

[48] Boulton AJ, Armstrong DG, Albert SF, Frykberg RG, Hellman R, Kirkman MS, et al ( 2008). Comprehensive foot examination and risk assessment: a report of the task force of the foot care interest group of the American Diabetes Association, with endorsement by the American Association of Clinical Endocrinologists. Diabetes care.

[49] Khalooei A, Meymand MM ( 2020). Frequency of Diabetes Foot Ulcer and Related Factors Among Adult Diabetic Patients in the Diabetes Center of Kerman, Iran. Semj.

[50] Golsha R, Baylari Z, Tajik M, Sohrabi A, Montazeri M ( 2020). Evaluation of Prognostic Risk Factors in Patients with Diabetic Foot Ulcers Admitted to Sayyad Shirazi Hospital in Gorgan during 2018-2020. Tabari Biomed Stu Res J.

[51] Sriyani KA, Wasalathanthri S, Hettiarachchi P, Prathapan S ( 2013). Predictors of diabetic foot and leg ulcers in a developing country with a rapid increase in the prevalence of diabetes mellitus. PLoS One.

[52] Maubon D, Garnaud C, Calandra T, Sanglard D, Cornet M ( 2014). Resistance of Candida spp. to antifungal drugs in the ICU: where are we now?. Intensive Care Med.

[53] Cabeceira HDS, Souza D, Juliano Y, Veiga DF ( 2019). Work ability and productivity in patients with diabetic foot. Clinics (Sao Paulo)..

[54] Saeed N, Nasir T, Burki B, Channa GA ( 2010). Mini-cholecystectomy: a feasible option. J Ayub Med Coll Abbottabad.

[55] Hurley L, Kelly L, Garrow AP, Glynn LG, McIntosh C, Alvarez-Iglesias A, et al ( 2013). A prospective study of risk factors for foot ulceration: the West of Ireland Diabetes Foot Study. QJM.

[56] Vibha S, Kulkarni MM, Kirthinath Ballala A, Kamath A, Maiya GA ( 2018). Community based study to assess the prevalence of diabetic foot syndrome and associated risk factors among people with diabetes mellitus. BMC Endocr Disord.

[57] Alam F, Islam MA, Gan SH, Khalil MI ( 2014). Honey: a potential therapeutic agent for managing diabetic wounds. Evid Based Complement Alternat Med.

[58] Zubair M, Ahmad J ( 2015). Plasma Heat Shock Proteins (HSPs) 70 and 47 levels in diabetic foot and its possible correlation with clinical variables in a North Indian Tertiary care hospital. Diabetes Metab Syndr.

[59] Caprnda M, Mesarosova D, Ortega PF, Krahulec B, Egom E, Rodrigo L, et al ( 2017 ). Glycemic Variability and Vascular Complications in Patients with Type 2 Diabetes Mellitus. Folia Med (Plovdiv)..

[60] Farooque U, Lohano AK, Hussain Rind S, Rind MS, Karimi S, Jaan A, et al ( 2020 ). Correlation of Hemoglobin A1c With Wagner Classification in Patients With Diabetic Foot. Cureus.

[61] Akash MSH, Rehman K, Fiayyaz F, Sabir S, Khurshid M ( 2020). Diabetes-associated infections: development of antimicrobial resistance and possible treatment strategies. Arch Microbiol.

[62] Raiesi O, Siavash M, Mohammadi F, Chabavizadeh J, Mahaki B, Maherolnaghsh M, et al ( 2017). Frequency of Cutaneous Fungal Infections and Azole Resistance of the Isolates in Patients with Diabetes Mellitus. Adv Biomed Res.

